# Reeling in countless effectors (RICE): time-course transcriptomics of rice blast disease reveal an expanded effector repertoire for *Magnaporthe oryzae*

**DOI:** 10.1093/plcell/koad035

**Published:** 2023-02-14

**Authors:** Bradley Laflamme

**Affiliations:** Assistant Features Editor, The Plant Cell, American Society of Plant Biologists, USA; Department of Molecular Genetics, University of Toronto M5R 0A3, Toronto, ON, Canada

Call them “empty carbs” if you want, but we owe them immensely: the major grass crops (maize, wheat, and rice) collectively account for about half of the global caloric intake. Unfortunately, our global dependance on grasses has inadvertently promoted some of the most destructive fungal phytopathogens, such as *Magnaporthe oryzae*, the causal agent of rice blast disease. The life cycle of *M. oryzae* occurs in three main steps spores land on rice plants, specialized cells called appressoria to penetrate host tissues, and then the pathogens thrive, spreads, and kill its host to feed before sporulating to spread further (Wilson and Talbot 2009). However, there remain several gaps in our knowledge about how the pathogen's virulence strategies evolve over the course of this dynamic infection cycle. The secretion of “effector” virulence proteins in host cells is known to underlie these virulence traits to some extent, but *M. oryzae*, like most fungal pathogens, has only had a portion of its effector repertoire revealed and appreciably studied in the context of its highly varied lifestyle.

**Figure 1.**
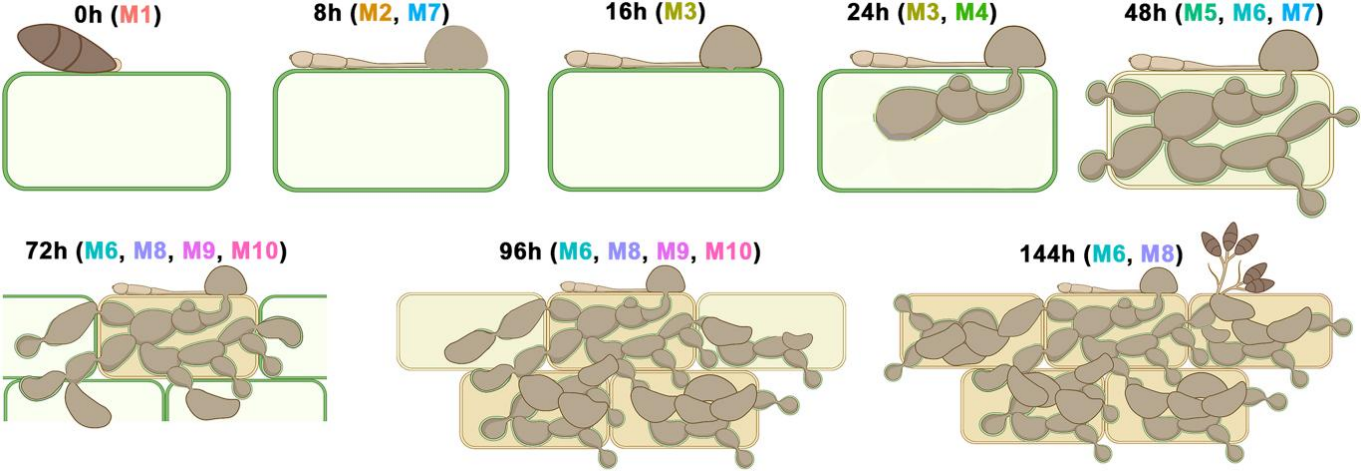
Schematic representation of the eight stages of *M. oryzae* infection used in this study, highlighting the gradual penetration, proliferation, and sporulation of *M. oryzae* over a 144-h period. The distinct gene co-expression modules relevant to each stage of infection are noted beside each timepoint. Reprinted from Yan et al. (2023), Figure 2B.

In this issue, Yan et al. (2023) take a holistic view of the *M. oryzae*-rice interaction using RNA-seq and offer the most comprehensive picture yet painted of the *M. oryzae* effector repertoire. While RNA-seq has previously been used to study *M. oryzae* disease development (Shimizu et al. 2019), Yan et al. made several adjustments to improve the resolution of fungal gene expression across the pathogen's multifaceted infection cycle. This included the use of multiple infection methods, two cultivars of varying sensitivity to *M. oryzae*, and eight time points covering a range of the most important stages in disease development (see Fig. 1).

Across a six-day infection time course, the group identified ten modules of co-expressed fungal genes that frequently corresponded to key phases in the pathogen's life cycle, such as appressorium development, necrotrophy, or conidiation. These modules captured not only many of the well-characterized genes involved in specific physiological processes (e.g. genes involved in appressorium development), but also the broader metabolic shifts that are known to occur over the course of *M. oryzae* infection, such as the switch toward secondary metabolism in the later necrotrophic stages.

Having established the stage-specific transcriptome of infecting *M. oryzae*, Yan et al. then set their sights on elucidating the entire effector repertoire of *M. oryzae—*that is, all the predicted secreted proteins that might be expressed throughout infection, which may interfere with host defenses and/or aid in an infection. They identified a whopping 863 differentially accumulated transcripts encoding predicted secreted proteins, with 546 being annotated as effectors. Based on structural predictions with AlphaFold and ChimeraX (Seong and Krasileva 2021), these 546 effectors were split into hundreds of structural clusters, suggesting their involvement in a wide range of biological processes. Several clusters had interesting expression signatures across the infection time course. For example, the well-studied MAX (*Magnaporthe oryzae* avirulence and ToxB-like) effectors, as well as putative ADP-ribosyltransferase effectors, both appeared to be upregulated only during certain stages of biotrophic development, suggesting these structurally distinct effectors have overlapping roles in virulence.

Yan et al. further characterized many of these putative effectors with an impressive array of functional assays. This included building effector-GFP fusions to assess subcellular localization and stage-specificity, validating the secretion of several effectors *in planta*, and showing that one novel effector, Mep1, confers a fitness advantage to *M. oryzae* during rice colonization. These experiments functionally validate the transcriptomic approach for the discovery of fungal effectors, while hinting at the hundreds of other possible functional pathways involved.

Standing alongside recent insights into the structure and evolution of fungal effectors (Seong and Krasileva 2023), this study by Yan et al. should invigorate fungal phytopathologists, who are becoming more and more invested in identifying and functionally cataloging effectors. The structural and regulatory complexity of fungal effectors has long made their study difficult relative to those of bacteria, but it is exciting to see that advances in genomics, transcriptomics, and structural biology may see fungi quickly catch up to the prokaryotes.
